# A Malignant Lymphoma Growing Inside a Cardiac Mixoma: A Case Report

**DOI:** 10.21470/1678-9741-2021-0081

**Published:** 2022

**Authors:** Sergio Pirola, Stefano Fiori, Fausto Maffini, Giulia Mostardini, Giorgio Mastroiacovo, Gianluca Polvani

**Affiliations:** 1 Department of Cardiac Surgery, Centro Cardiologico Monzino, IRCCS, Milan, Italy.; 2 Division of Diagnostic Haematopathology, European Institute of Oncology, IRCCS, Milan, Italy.; 3 Division of Pathology, European Institute of Oncology IRCCS, Milan, Italy.; 4 Department of Cardiovascular Sciences and Community Health, University of Milan, Italy.

**Keywords:** Myxoma, Lymphoma Heart Neoplasms, Epstein-Barr Virus, Postoperative Period

## Abstract

**Introduction:**

Lymphomas arising from cardiac myxomas represent a particularly rare pathology, with only few cases reported in the literature.

**Case presentation:** We report a complete excision of a malignant lymphoma arising from a cardiac myxoma in a 44-year-old female patient. The myxoma presented like a floating mass within the left atrium with a maximum diameter of 3.5 cm. The clinical post-operative period was uneventful and the patient was dismissed on the 6^th^ post-operative day.

**Conclusion:**

This case reinforces the concept of radical excision of cardiac neoplasms.

**Table:** 

Abbreviations, acronyms & symbols	
CCS	= Canadian Cardiovascular Society
CT	= Computed tomography
DLBCL-NOS	= Large B-cell lymphoma not otherwise specified
EBV	= Epstein-Barr virus
EBER	= Epstein-Barr virus-encoded small RNAs
FA-DLBCL	= Fibrin-associated diffuse large B-cell lymphoma
LMP1	= Latent membrane protein 1
NYHA	= New York Heart Association

## INTRODUCTION

Cardiac myxomas account for the majority of cardiac tumours (50%) and mainly affect middle-aged women^[1]^. Primary cardiac lymphoma is a very rare neoplasm that accounts for 2% of all primary cardiac tumours^[2]^. Lymphomas growing in the context of cardiac myxomas are extremely rare mixed tumours, with just few cases reported in literature.

### Case Presentation

A previously healthy 44-year-old female patient presented to our hospital from an outpatient cardiology clinic with an echocardiographic finding of a floating mass within the left atrium.

She was symptomatic for a few months for asthenia, dyspnoea and dizziness. She denied lipothymia or syncope and any history of drug/alcohol abuse or recent travel. Her family history was negative for cardiovascular/neoplastic diseases.

The patient was in good clinical condition, NYHA class I and CCS 0. Cardiac auscultation revealed a classical diastolic “tumour plop”, not accompanied by another significant murmur.

**Fig. 1:**
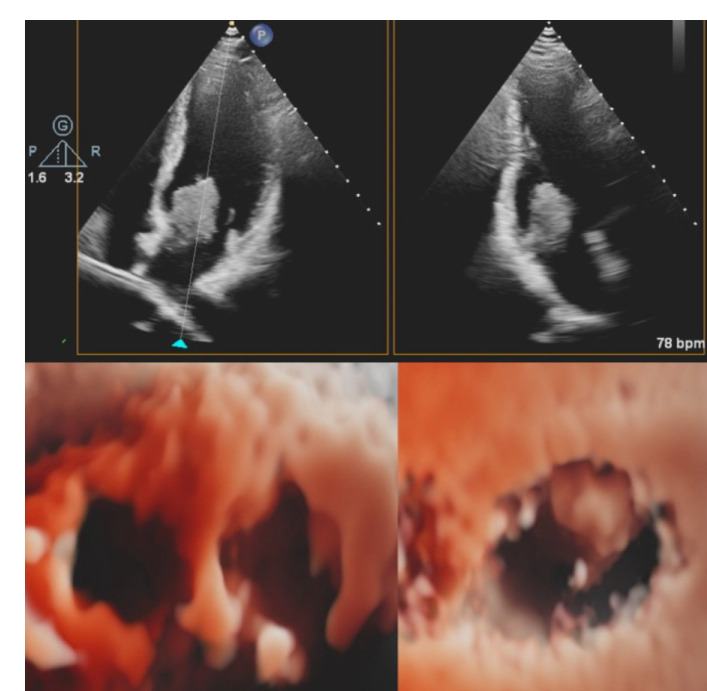
2D transthorachic echocardiography images and 3D reconstruction of the left atrial mass.

An urgent transthoracic echocardiogram with 3D digital reconstruction of the images was performed and confirmed the primary diagnosis (Figure 1). The mass originated from the fossa ovalis on the left side of the interatrial septum.

The maximum diameter was 3.5 × 3 cm. The mass had a lobulated, polypoid architecture and a narrow implantation stalk, appearing to be unstable, and prolapsed across the mitral valve orifice during diastole.

**Fig. 2:**
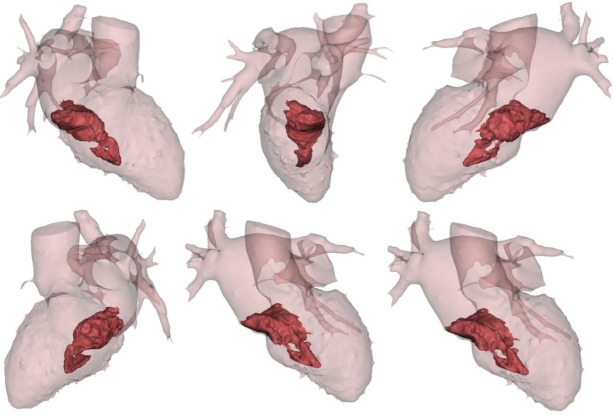
CT-based 3D virtual reconstruction of the mass performed using MIMICS software showing the extremely irregular borders and a narrow implantation stalk in the fossa ovalis.

To complete the diagnostic framework, cardiac, lung and brain computed tomography (CT) scans (Figure 2) were performed, without any pathological findings and recent cardio-embolic events were also excluded.

The patient was then scheduled for surgery. The operation was performed trough a left atriotomy. The tumour presented as a pale pink, grape-like, semi-transparent mass. It had a particularly gelatinous and friable consistency, with a high risk of fragmentation during surgical manipulation. Due to these atypical characteristics, we suspect the malignant nature of the neoplasm.

To perform a cautious removal of the mass, we started resecting it 2 mm from the implant base using a No. 11 scalpel blade to reduce the risk of tumour fragmentation.

The next step was to perform a radical full-thickness resection of the implant base, located in the atrial septum, leaving a safety margin of 5 millimetres. This was followed by repeated and extended washing of the left chambers of the heart to reduce the risk of embolization of the remaining tumour fragments. The redundant interatrial septum, though largely resected, was closed by a direct suture. No complications occurred in the postoperative period, and the patient was discharged on the 6^th^ post-operative day to a rehabilitation facility.

At gross examination, surgical specimens included: a lesion with dimensions of 3.5 × 3 cm, pedunculated, translucid and with a villous surface, focally covered by fibrinous exudate; a 1.5 cm implantation stalk and a fragment of interatrial septum.

**Fig. 3:**
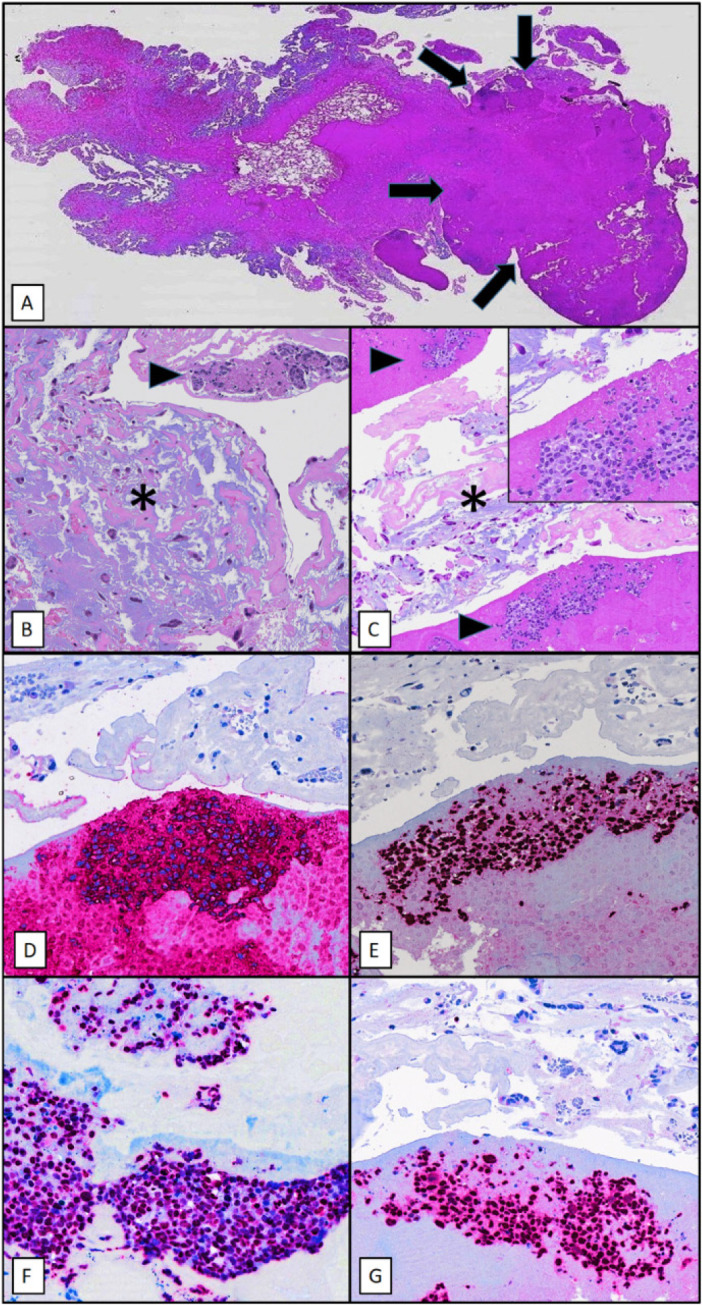
A, B, C: Morphological features of the lesion. Whole-mount section of myxoma, with villous surface and a superficial fibrinous cap (arrows) containing lymphoid aggregates (A, haematoxilin-eosin). The myxoma consists of a translucent loose matrix containing scattered stellate cells (asterisk). The lymphoid cells embedded in fibrinous material (arrowhead) are large-sized and atypical (B, C, inset, haematoxilin-eosin). D, E, F: Immunophenotypic features of fibrin-associated diffuse large B-cell lymphoma. The atypical lymphoid cells have a B-cell phenotype, being positive for CD20 (A) and PAX5 (B), are diffusely positive for EBV/EBER (C) and have a high Ki67 proliferation index (D), in contrast to the myxoma cells (D, upper half), which are almost completely

Histological analysis showed that the lesion consisted of myxoid matrix containing thin vessels and scattered stellate cells, without mitotic activity and cytologic atypia. Interestingly, the fibrinous layer focally covering the lesion contained few dense aggregates consisting of large, blastic lymphoid cells, with clear-cut atypical features (Figure 3).

On immunohistochemical staining, the stellate cells embedded in the myxoid matrix were positive for CD34 and negative for cytokeratins AE1/AE3, CD31 and CD45/LCA, and with a very low Ki67 proliferation rate (<2%). Such findings were consistent with cardiac myxoma.

Further immunohistochemical evaluation showed a B-cell phenotype (positive for CD20, PAX5, CD79A) of the atypical, blastic lymphoid cells, along with partial positivity for BCL6, IRF4, BCL2, CD30 and C-MYC (<40% of cells), negativity for CD10, CD5, CD15, ALKc and HHV8, and a very high Ki67 proliferation index (>80%). *In situ* hybridization for Epstein-Barr virus (EBV/EBER) was diffusely positive (Figure 3).

Histological features and incidental findings were all consistent with an EBV-positive fibrin-associated large B-cell lymphoma, arising within a cardiac myxoma.

The implantation stalk was focally involved by myxoma, but free of lymphoma, suggesting complete resection of the lesion.

Total body CT scan and 18-FDG positron emission tomography excluded distant metastases. Oncologic consultation did not indicate adjuvant chemotherapy or radiotherapy.

## DISCUSSION

Myxoma is the most common type of tumour among all primary cardiac neoplasms^[1]^. They usually originate from the fossa ovalis with a growth directed towards the left atrial cavity^[3]^. Common symptoms are related to embolic events due to myxoma fragments embolization and syncope episodes due to diastolic mitral valve obstruction^[1]^.

Cardiac lymphomas are instead rare, amounting to only 0.6% of all cardiac tumours^[4]^. Although several subtypes of lymphomas may arise in the heart, the most common is diffuse large B-cell lymphoma not otherwise specified (DLBCL-NOS), usually associated with an aggressive biological behaviour^[5-7]^. On the other hand, the most frequent lymphoproliferative disease associated with cardiac myxoma is fibrin-associated diffuse large B-cell lymphoma (FA-DLBCL), with 16 cases reported so far^[8]^.

FA-DLBCL is a recently recognized entity^[9]^, included within the spectrum of diffuse large B-cell lymphomas associated with chronic inflammation and has highly distinctive features: it arises in fibrinous material covering or contained in sites of chronic inflammation (most commonly within the cardio-vascular system), neither forming a mass nor producing specific symptoms^[9,10]^. Typically, the diagnosis is incidental, during histologic examination. FA-DLBCL is invariably EBV-positive.

It is therefore likely that most of the previously reported cases of lymphomas arising in association with cardiac myxomas would be re-classified to FA-DLBCL according to the current classification of the World Health Organization, due to the concomitant^[9]^ positivity for EBV^[10-17]^ and for the typical location within the fibrinous material covering the myxoma^[18,19]^.

More importantly, despite aggressive histological features (large cell morphology, high proliferation rate), FA-DLBCL is a localized disease with an indolent behaviour and a favourable outcome even with surgical excision alone. The rare event of relapse and the associated risk of local invasion^[3,9,20]^, however, reinforce the need for a complete surgical excision.

## CONCLUSION

Our case report suggests that cardiac surgeons must be aware that even apparently benign cardiac masses can conceal malignant lesions, adopting a presumptive attitude of “malignancy until proven otherwise”, which should prompt always an accurate and radical excision of the mass.

Recognition of a macroscopic unusual aspect of the lesion is of paramount importance, as all reported cases of lymphoid proliferation arising within a cardiac myxoma showed a partial or global gelatinous aspect of the neoplasm (especially in its peripheral areas) (Table 1).

**Table 1:** Available literature data regarding cases of myxomas in which B-cell lymphomas were identified.

First author	Location and size	Symptoms	Lymphoma	EBV type	Gross analysis	Follow-up
Bagwan^[17]^	Left atrium (4 × 2 cm)	Multiple strokes	Diffuse large B-cell lymphoma	Not available	Gelatinous, grape-like, friable	Not available
Dimitrova^[18]^	Left atrium (7.5 × 4.5 cm)	Chest pain	Diffuse large B-cell lymphoma	Not available	Partially fimbriated surface, gelatinous and necrotic parts	Not available
Loong^[11]^	Left atrium (6.5 × 4 cm)	Cardiogenic shock, ischemic stroke	Diffuse large B-cell lymphoma	Type III latency (EBER+, LMP1+)	Not described	Patient deceased for chemotherapy complications (neutropenia + pneumonia) at 5 months
Svec^[12]^	Left atrium (3.7 × 1.5 cm)	Ischemic stroke	Diffuse large B-cell lymphoma	Type III latency (EBER +, LMP1+)	Not described	No evidence of disease at 7 months
Bartoloni^[13]^	Left atrium (5.5 × 4.5 cm)	Fever and progressive fatigue	Atypical lymphoid B-cell proliferation	Type II latency (EBER +, LMP1+)	Glistening, villous and gelatinous surface	No evidence of disease at 72 months
Tapan^[15]^	Left atrium (4.2 × 3.6 cm)	Palpitations	Diffuse large B-cell lymphoma	Type III latency (EBER +, LMP1+)	Not described	No evidence of disease at 12 months
Aguilar^[14]^	Left atrium (6 × 2.5 cm)	Transient ischemic attack	Diffuse large B-cell lymphoma	Type III latency (EBER +, LMP1+)	Not described	No evidence of disease at 42 months
Pineda^[3]^	Left atrium (6.5 × 3 cm)	Coronary embolization	Diffuse large B-cell lymphoma	Not available	Reddish, gelatinous	Not available
Park^[20]^	Left atrium (6 × 3.2 cm)	Peripheral arterial embolization	Diffuse large B-cell lymphoma	Type III latency, EBER +	Irregular surface, friable	Not available
Boyer^[9]^	Left atrium (? cm)	Syncope	Diffuse large B-cell lymphoma	EBER+	Not available	No evidence of disease at 130 months
Boyer^[9]^	Left atrium (? cm)	Syncope, cough, dyspnoea	Diffuse large B-cell lymphoma	EBER+, LMP1+	Reddish, gelatinous	Patient died at 2 months for cardiac cause
Boyer^[9]^	Left atrium (? cm)	Dyspnoea, respiratory failure	Diffuse large B-cell lymphoma	EBER+, LMP1+	Not available	Recurrent FA-DLBCL at mitral valve after 25 months. Patient died at 26 months (embolic stroke).
Yan^[10]^	Left atrium (? cm)	Congestive heart failure	Diffuse large B-cell lymphoma	Type III latency, EBER+, LMP1+	Myxoid appearance with an abundant fibrinous or mucinous background in the centre	No evidence of disease at 7 months
Yan^[10]^	Left atrium (? cm)	Congestive heart failure	Diffuse large B-cell lymphoma	Type III latency, EBER+, LMP1+	Myxoid appearance with an abundant fibrinous or mucinous background in the centre	No evidence of disease at 84 months
Yan^[10]^	Left atrium (? cm)	Congestive heart failure	Diffuse large B-cell lymphoma	Type III latency, EBER+, LMP1+	Myxoid appearance with an abundant fibrinous or mucinous background in the centre	No evidence of disease at 3 months
Yan^[10]^	Left atrium (? cm)	Congestive heart failure	Diffuse large B-cell lymphoma	Type III latency, EBER+, LMP1+	Myxoid appearance with an abundant fibrinous or mucinous background in the centre	No evidence of disease at 120 months
Present case	Left atrium (3.6 × 3 cm)	Asthenia, dyspnoea and dizziness	Diffuse large B-cell lymphoma	EBER+, LMP1+	Gelatinous, grape-like, pale pink, semi-transparent, extremely friable	No evidence of disease at 4 months

EBER=Epstein-Barr virus-encoded small RNAs; LMP1=latent membrane protein 1

The rarity of this disorder is an obstacle to prospective studies. Endocavitary masses with the peculiar macroscopic characteristics we have described could indicate a high probability of lymphocytic infiltration in the context of myxomatous masses. In this case, a specific, integrated diagnostic and therapeutic pathway should be followed, including: wide and radical resection and possibly a bone marrow specimen (easily collected during surgery from the sternal bone), to ensure a fast staging if the lesion would be proven malignant.

**Table:** 

Authors' roles & responsibilities	
SP	Substantial contributions to the conception or design of the work; or the acquisition, analysis, or interpretation of data for the work; drafting the work or revising it critically for important intellectual content; final approval of the version to be published
SF	Histological analysis of the surgical specimen, curation of the anatomopathological part of the paper
FM	Histological analysis of the surgical specimen, curation of the anatomopathological part of the paper.
GM	Radiological image processing and reconstruction with special editing software (MIMICS, Materialise)
GM	Collaboration in writing the paper and proofreading it, as well as editing the literature review
GP	Coordination and supervision of the entire work of the team of researchers and final endorsement to the publication of the paper
